# Slippery Surface Based on Photoelectric Responsive Nanoporous Composites with Optimal Wettability Region for Droplets' Multifunctional Manipulation

**DOI:** 10.1002/advs.201801231

**Published:** 2018-11-20

**Authors:** Keyu Han, Liping Heng, Yuqi Zhang, Yao Liu, Lei Jiang

**Affiliations:** ^1^ Key Laboratory of Bio‐Inspired Smart Interfacial Science and Technology of Ministry of Education Beijing Key Laboratory of Bio‐inspired Energy Materials and Devices School of Chemistry Beihang University Beijing 100191 China; ^2^ College of Chemistry and Chemical Engineering Yan'an University Yan'an Shaanxi 716000 P. R. China

**Keywords:** droplet motion, patterned writing, photoelectric cooperative, slippery surfaces, wettability region

## Abstract

The development of responsive slippery surfaces is important because of the high demand for such materials in the fields of liquid manipulation on biochips, microfluidics, microreactions, and liquid‐harvesting devices. Although great progress has been achieved, the effect of substrate wettability on slippery surfaces stability is overlooked by scientists. In addition, current responsive slippery surfaces generally function utilizing single external stimuli just for imprecisely controlling liquid motion, while advanced intelligences are always expected to be integrated into one smart interface material for widespread multifunctional applications. Therefore, designing slippery surfaces that collaboratively respond to complex external stimuli and possess sophisticated composite function for expanding applications from controlling droplets motion to patterned writing is urgently needed but remains a challenge. Here, a photoelectric cooperative‐responsive slippery surface based on ZnO nanoporous composites is demonstrated. First, the effect of composite surface wettability on slippery surface stability is systematically researched and the optimum wettability region for fabricating stable slippery surfaces is determined. Furthermore, controllable droplet motion and patterned writing are realized on the same slippery surfaces under photoelectric cooperative stimuli, and the related response mechanism is also deeply studied. This kind of material has potential applications in biochips, microfluidics, in situ patterning, and water‐harvesting systems.

Slippery surfaces have recently been extensively studied^1^, ^2^, ^3^, ^4^ because of their excellent self‐cleaning properties, self‐healing properties after being scratched or damaged,[[qv: 2c,4e,5]] and super‐repellency against various liquids.[[qv: 2a,3d,g,4f,i]] Current studies in this field mainly focus on three aspects: (1) preparing slippery surfaces using different substrates, including porous Teflon membranes,^6^ honeycomb polymer films,[[qv: 2b]] electrodeposited nanoporous tungstite films,[[qv: 2c]] inverse colloidal monolayers,[[qv: 4e]] 3D cross‐linked oil gel system,[[qv: 3b,h,l,7]] covalently attached omniphobic coatings,^8^ and lubricants such as perfluorinated liquids,[[qv: 2b,c,4e,6]] silicone oil,[[qv: 1a,3b,4k]] and ionic liquids;[[qv: 3f]] (2) understanding the theoretical design principles for slippery surfaces;[qv: 9] and (3) expanding the new application of these surfaces in the anti‐icing,[[qv: 3i,k,l]] antifouling,[[qv: 3e,n,4a–d,k,10]] anticorrosion,^11^ drag reduction,^12^ liquid separation,^13^ and dropwise condensation fields.[[qv: 1c,3m,14]] Smart slippery surfaces, as emerging representatives of advanced interface materials, have been developed for controlling liquid drop motion under the stimuli of external fields such as mechanical forces,[[qv: 2b,3a]] magnetic field,^15^ temperature,[[qv: 3h,16]] and electric field.[qv: 17]

Taking these facts into account, the substrates used for preparing slippery surfaces generally contain two types of materials: one is a directly utilized low‐surface‐energy material such as polytetrafluoroethylene,[[qv: 3a,4b]] poly(vinylidene fluoride‐*co*‐hexafluoropropylene),[qv: 18] poly(butyl methacrylate‐*co*‐ethylene dimethacrylate),[[qv: 4c,d]] polypropylene,[[qv: 16c]] or polydimethylsiloxane,[[qv: 3h,7]] and the other is a high‐surface‐energy material such as TiO_2_,[qv: 19] AlO(OH),[[qv: 4f]] Al[[qv: 3i]] or SiO_2_,[[qv: 3n,5]] which must be modified with low‐surface‐energy molecules of perfluorinated silane,[[qv: 2c,4h,6]] chlorosilane,[[qv: 3j,16a]] aliphatic acids,[qv: 20] and polymer molecular brushes[[qv: 8b,19,21]] to reduce the surface energy for fabricating slippery surfaces.[qv: 6,22] Although great progress has been made in preparing slippery surfaces, the relationship between substrate surface energy, which can be indicated by substrate wettability, and slippery surface stability has not been clearly studied but is of vital importance for guiding the preparation of stable slippery surfaces. Furthermore, current reported responsive slippery surfaces generally utilize single external stimulus for controlling liquid motion; Usually, the response speed is slow, and the motion behavior of the liquid is hard to be precisely controlled. As a more effective control strategy, the preparation of slippery surfaces that synergistically respond to two or more complex external stimuli is still in its infancy.^23^ In addition, with the development of smart interface materials, various advanced intelligences are expected to be integrated into one material to achieve multifunctional applications. Therefore, designing slippery surfaces that collaboratively respond to multiple external stimuli and possess sophisticated composite function was urgently needed but remains a challenge.

In this paper, we presented photoelectric cooperative‐responsive ZnO‐*cis*‐bis(4,4′‐dicarboxy‐2,2′‐bipyridine) dithiocyanato ruthenium(II)‐heptadecafluorodecyl‐trimethoxysilane (ZnO‐N3‐FAS) slippery surfaces, which were fabricated through hydrothermal synthesis and subsequent photosensitization with a dye and hydrophobization with a low‐surface‐energy silane; finally, a lubricating liquid (silicon oil) filled the composites. First, we systematically studied the influence of substrate wettability on the formation of slippery surfaces and determined the optimum wettability region for presenting a stable slippery performance. Second, photoelectric cooperative‐responsive behavior and the droplets' multifunctional manipulation including controllable droplet motion and patterned writing were achieved on the slippery surface. Additionally, we studied their profound photoelectric cooperative response mechanism. This work promotes understanding the relationship between substrate wettability and the stability of slippery surfaces and the underlying principles of the photoelectric cooperative response on slippery surfaces, providing a promising material for applications in biochips, microfluidic systems, controllable drug‐delivery, and in situ patterning.

To prepare photoelectric cooperative responsive slippery surfaces, a substrate with photoconductive properties and a porous structure are necessary.^24^ Aligned ZnO nanorod array with a porous structure, which is wide‐band‐gap semiconductor and exhibits photoconductive properties under illumination, is an ideal candidate. The aligned ZnO nanorod array is first grown perpendicularly onto the substrate and then sensitized with an N3 dye as a composite photoconductor layer. Finally, the N3‐coated ZnO‐nanorod array is modified by FAS to reduce the surface energy, that is, improve the surface hydrophobicity. **Figure** **1** shows the top‐view and side‐view scanning electron microscopy (SEM) images and the schematic diagrams of the as‐prepared aligned ZnO (uncoated, N3‐coated, N3‐coated and FAS‐modified) nanorod arrays. Figure 1a clearly indicates that the original ZnO‐nanorod arrays were grown almost perpendicularly onto the substrate (gray pillar in schematic diagrams), with an average diameter of 69.6 ± 11.8 nm and an average length of 1.61 ± 0.07 µm. X‐ray diffraction (XRD) pattern with a remarkable (002) peak (Figure S1, Supporting Information) confirmed that the ZnO (0001) planes are oriented parallel to the indium tin oxide (ITO) surface. When the ZnO nanorods were coated with N3 (red border in the schematic diagrams of Figure 1b; Figure S2, Supporting Information) and furtherly modified by FAS (blue border in schematic diagram of Figure 1c; Figure S2, Supporting Information), the morphologies did not change significantly (Figure 1b,c). The average diameter increased from 69.6 ± 11.8 nm to 72.1 ± 9.9 nm, and 79.4 ± 7.6 nm, respectively, and the final average spacing between composite nanorods was 76.3 ± 20.9 nm; the nanorod length keeps almost the same. SEM‐energy dispersive X‐ray spectroscopy (EDS) element analysis (Figure S3, Supporting Information) clearly indicate that the N3 and FAS have been successfully coated onto the underlying ZnO‐nanorod surfaces with uniform distribution. Figure S4a of the Supporting Information shows the wettability of the different substrates measured by water contact angles (CA) on as‐prepared (uncoated, N3‐coated, N3‐coated and FAS modified) surfaces, which were 23.2° ± 1.2°, 33.2° ± 1.3°, and 133.2° ± 1.7°, indicating that the N3 sensitizer and FAS modification increased the hydrophobicity of the surface.

**Figure 1:**
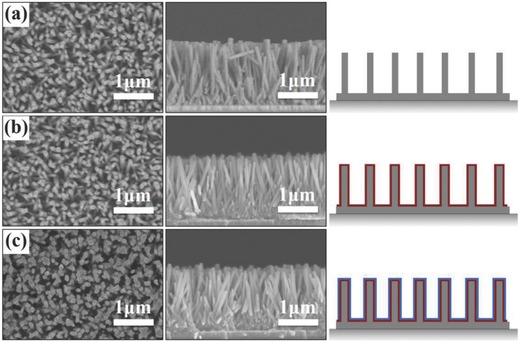
Top‐view and side‐view SEM images and schematic diagrams of as‐prepared a) original ZnO nanorod array with a rod diameter of 69.6 ± 11.8 nm, b) N3‐coated ZnO nanorod array with a rod diameter of 72.1 ± 9.9 nm, and c) N3‐coated ZnO nanorod array modified by FAS with a rod diameter of 79.4 ± 7.6 nm. The nanorods (gray pillars in schematic diagrams) are grown almost perpendicularly onto the substrate, with a length of 1.61 ± 0.07 µm, and the N3 (red border in schematic diagram of (b) and FAS (blue border in schematic diagram of (c) modification slightly increases the diameters of the ZnO nanorods.

For the construction of slippery surfaces, the aligned ZnO‐nanorod arrays provide a substrate with many nanogaps to hold lubricating liquid. However, the ideal slippery surface with a molecular‐level flat interface should satisfy an essential substrate wettability condition^6^, ^22^ because inappropriate substrate wettability would make the repelling droplet sink in the lubricating layer and directly stick to the underlying high‐surface‐energy substrate, which would cause the surface to lose its slippery property. What kind of wettability region should the composite surface possess for achieving a stable slippery performance? To answer this question, we systematically studied the effect of substrate wettability on the formation of the slippery surface. Generally, if a liquid drop floats on the slippery surface, the combination surface tensions (∆γ) of the test liquid drop, lubricating fluid and substrates should satisfy the following criteria[[qv: 9b,13b,22]]

(1) $$\Delta \gamma =\gamma_{1}\cos\theta_{1}-\gamma_{2}\cos\theta_{2}-\gamma_{12}>0$$

where γ_1,_γ_2_, and γ_12_ are the surface tensions of the test liquid drop (water, γ_1_ = 72.7 mN m^−1^) and lubricating liquid (silicon oil, γ_2_ = 17.6 mN m^−1^) and the interfacial tension between them (γ_12_ = 56.5 mN m^−1^), respectively, and θ_1_ and θ_2_ are the CAs of the test liquid and lubricant on the composite surface, respectively. In the above criteria, an assumption of the working conditions is that the lubricant layer covers the surface features.[qv: 6] To meet this assumption, all slippery films used in this work were prepared by immersing the film in silicone oil for 3 min to ensure sufficient lubricant on the substrate. We precisely regulated the surface wettability of ZnO‐nanorod arrays from mildly hydrophobic to superhydrophobic using different modification conditions (Figure S2, Supporting Information). A series of CAs of water and silicon oil on these composite surfaces were obtained (**Table** **1**). The ∆γ parameter has been calculated based on Equation (1) using the measured CAs and the related surface tensions of water, silicon oil and water/silicon oil (Table 1). According to the water/silicon oil CAs as a function of ∆γ, the ZnO surface wettability status can be divided into three regions by the black dotted line (∆γ = 0) and blue dotted line (dewetting) (**Figure** **2**a). The results clearly showed that ∆γ will be lower than 0 when the water CA is below ≈125.7° ± 2.3° (region I). In this region, owing to the low silicon oil CAs (smaller than 34.8° ± 3.0°) and large capillary forces of the nanorod array surface, the lubricating liquid can fully penetrate the nanotexture and shows excellent stability even after 24 h, which was confirmed by the optical photographs of black‐dyed silicon oil on the composite surface (Figure S5a, Supporting Information and top inset in region I of Figure 2a). However, water droplets sink in the oil layer and stick to the rough ZnO substrate in this case because ∆γ < 0 in this region.^22^ A contact model schematic diagram is shown in the left section of Figure 2b; a water droplet (blue part) directly contacts both the ZnO nanorods (gray pillars) and silicon oil (red part) in this region. Thus, this water droplet cannot slip on the composite surface, even when the substrate tilted angle (TA) reaches 90° (bottom inset optical photographs in region I of Figure 2a). This result can be attributed to the insufficient hydrophobicity of ZnO, which adsorbs more strongly to the water than to the oil. In region II, 125.7° ± 2.3° < water CAs < 140.7° ± 1.3°, 34.8° ± 3.0° < silicon oil CAs < 41.3° ± 3.2°, and ∆γ > 0, indicating that silicon oil can not only infiltrate into the nanorod arrays to form a stable lubricating layer, even after 24 h (digital photographs of black‐dyed silicon oil on the composite surface in Figure S5b of the Supporting Information and top inset in region II of Figure 2a), but also support the water droplets on its surface without them directly contacting the ZnO arrays; the contact model schematic diagram is shown in the middle section of Figure 2b. In this case, water can slip easily on slippery surfaces and exhibits a small water sliding angle (SA) of ≈4.1° ± 1.4° (bottom inset optical photographs in region II of Figure 2a). This behavior can be attributed to the proper surface energy of the composite surface, which ensures that the water droplet (blue part) floats on the surface of the silicon oil (red part) and cannot contact the underlying ZnO surface. Further decreases in surface energy to region III (water CAs > 140.1° ± 3.2° and silicon oil CAs > 41.3° ± 3.2°), even though ∆γ is also above 0, causes clear dewetting of the lubricant (Figure S5c of the Supporting Information and top inset digital photographs in region III of Figure 2a). In this case, the silicon oil cannot fully fill in the gaps of ZnO nanorods because of their large oil CA values, and the wettability model schematic diagram is shown in the right section of Figure 2b. Therefore, the composite surfaces in region III are also not suitable for fabricating a stable slippery surface despite ∆γ being greater than 0. The above result showed that region II (125.7° ± 2.3° < water CAs < 140.7° ± 1.3° and 34.8° ± 3.0° < silicon oil CAs < 41.3° ± 3.2°) is a suitable working region for preparing stable slippery surfaces. In this region, water SAs on slippery surfaces of ≈4.1° ± 1.4° do not change with a variation in surface wettability (Figure 2c). Hence, composite surfaces with a water CA of ≈133.2° ± 1.7° (Figure S4a, Supporting Information) were used for the following experiment.

**Table 1:** CAs of water (θ_1_) and silicon oil (θ_2_) on different ZnO‐N3‐FAS surfaces and the calculated Δγ value

Sample no.	θ_1_ [°]	θ_2_ [°]	∆γ [mN m^−1^]
1	84.0 ± 2.2	10.7 ± 2.8	−46.8
2	94.5 ± 2.4	12.6 ± 2.6	−33.6
3	105.9 ± 2.2	26.9 ± 1.6	−20.8
4	115.7 ± 2.0	31.3 ± 1.3	−10.0
5	122.8 ± 2.1	33.1 ± 2.1	−2.4
6	123.8 ± 3.7	34.5 ± 2.6	−1.8
7	125.7 ± 2.3	34.8 ± 3.0	0.38
8	131.1 ± 3.1	36.2 ± 2.3	5.5
9	137.6 ± 3.2	37.8 ± 2.9	11.1
10	140.1 ± 3.2	41.3 ± 3.2	12.5
11	144.1 ± 3.3	44.7 ± 3.1	15.0
12	148.9 ± 3.9	46.1 ± 2.7	18.0
13	150.4 ± 4.9	48.7 ± 4.3	18.3
14	155.0 ± 6.9	54.8 ± 5.7	19.5

**Figure 2:**
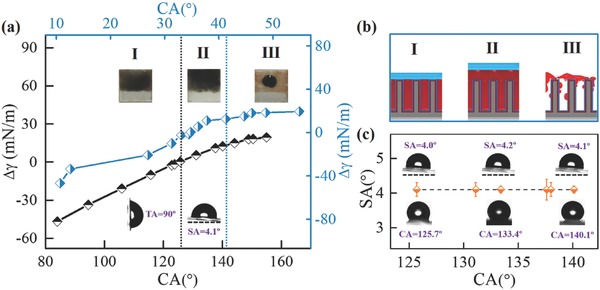
Influence of surface wettability on the formation of slippery surfaces. a) Water/silicon oil CAs versus *∆γ* based on the equation ∆γ = γ_1_ cosθ_1_ – γ_2_ cosθ_2_ – γ_12_. Surface wettability status can be divided into three regions by the black dotted line (*∆γ* = 0) and the blue dotted line (dewetting). In region I, for low silicon oil CAs (smaller than 34.8° ± 3.0°) and large capillary forces of the nanorod surface, the lubricant can easily infiltrate into the nanotexture with excellent stability, even after 24 h (top inset photographs in region I). However, water droplets sink into the oil layer and firmly stick to the ZnO substrate for *∆γ* < 0 (bottom inset SA photographs). In region II, (125.7° ± 2.3° < water CA < 140.7° ± 1.3°, 34.8° ± 3.0° < silicon oil CA < 41.3° ± 3.2° and *∆γ* > 0), silicon oil not only penetrates the nanorod arrays (top inset photographs) but also supports water, allowing it to readily slip on the composite surface with a small SA of ≈4.1° ± 1.4° (bottom inset CA photographs). In region III, (water CA > 140.1° ± 3.2° and silicon oil CA > 41.3° ± 3.2°), even though *∆γ* > 0, dewetting of the lubricant clearly occurred. b) Wettability model schematic diagrams corresponding to the above three regions. c) Water SA on slippery surfaces versus water CA on N3‐FAS‐modified ZnO surface in region II. The results clearly indicate that the water SA was unchanged with the variation of substrate surface wettability.

CA and SA variations of the slippery surface induced by electric field and photoelectric cooperative stimuli were systematically studied (**Figure** **3**a–c; Figure S6, Supporting Information). The conductive copper wire in the experimental device (Figure 3a), which was used to apply voltage, increased the resistance to the droplet sliding by producing an additional *f* (frictional force between the liquid droplet and the copper wire). Hence, the slippery surface presented a water SA of ≈13.3° ± 1.2° (Figure 3b) at the initial stage, greater than that without a copper wire (≈4.1° ± 1.4°) (Figure 2c). When a voltage is applied to the water without illumination, the water SA began to increase after 8.0 V and finally reached 89° ± 0.9° at 13.0 V (Figure 3b). In addition, the water droplet was firmly pinned on the film at voltages greater than 13.0 V. Analogously, with 80 mW cm^−2^ illumination, the water SAs started to increase when the voltage was greater than 4.0 V and finally reached 88.5° ± 1.5° at 8.0 V. Similarly, the water droplet was irreversibly pinned on the slippery surfaces when the voltage exceeded 8.0 V. The water CA changes on the slippery surfaces transitioned at the same voltages as the water SA, with and without light illumination (Figure S6a, Supporting Information). Based on the CA and SA versus voltage curves shown in Figure S6a (Supporting Information) and Figure 3b, the effects of the applied voltage can be divided into three regions. In region I (0.0–4.0 V), the SA does not change regardless of illumination because the applied voltage does not reach the threshold voltage (bias voltage at which the water CA and SA start to change); in region II (≈4.0–8.0 V), the SA increases under light illumination and does not change without illumination because the light illumination reduces the threshold voltage; in region III (≈8.0–13.0 V), the SA increases even without light illumination because the applied voltage surpasses the threshold voltage. Thus, region II is the suitable working region for photoelectric cooperative control of the droplet motion, and region III is the suitable working region for photoelectric cooperative patterned writing. Furthermore, the effect of light intensity on photoelectric cooperative SA and CA variation was investigated at a fixed voltage of 6.0 V (Figure S6b, Supporting Information), and the results clearly indicated that the CA gradually decreases and the SA increases with increasing light intensity. In addition, reversible water SA switching on the slippery surface under alternating on‐off illumination was achieved at an applied voltage of 6.0 V (Figure 3c). In this case, water droplets slide with an SA of ≈13° without illumination and ≈58° with 80 mW cm^−2^ illumination. The films exhibited durable reversible SA switching after even five cycles. These results have strongly demonstrated that a photoelectric cooperative‐responsive SA transition can be realized on the slippery surface in region II.

**Figure 3:**
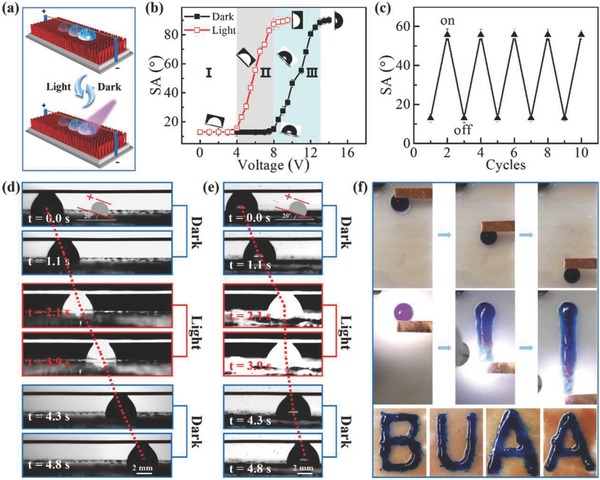
Water SA variation on the slippery surfaces under electric fields and photoelectric cooperative stimulus: a) schematics of the SA measurement on the slippery surface (red color) under applied voltage with and without illumination. b) SA as a function of the applied voltage measured with (□) and without (■) illumination (80 mW cm^−2^). The effects of the applied voltage can be divided into three regions. In region I, SAs do not change with increasing applied voltage, even with illumination; in region II, SAs increase with increasing applied voltage under illumination but remain unchanged without illumination; in region III, SAs change with increasing applied voltage, even without illumination. c) The SAs reversibly switch in response to a light alternating on and off (80 mW cm^−2^) at a 6.0 V voltage. Smart manipulation of droplets on the slippery surfaces: d) In region I, a water droplet (2 µL) can slide regardless of illumination because the applied voltage (2.0 V) does not reach the threshold voltage. e) In region II, droplet (2 µL) motion on the slippery surfaces was easily controlled through the photoelectric (6.0 V and 80 mW cm^−2^) cooperative stimuli. f) In region III, the water droplet can easily slide on the surfaces without any trace under a 10 V voltage (top image). When illumination (80 mW cm^−2^) and 10.0 V were applied simultaneously, photoelectric wetting occurred, and patterned line and letters (middle and bottom image) were successfully written by the “copper pen.”

One of the significant goals of this work is to achieve the smart manipulation of droplets. Based on the above three divided regions, water droplet sliding regulation and patterned writing were demonstrated as follows (Figure 3d–f). A 2 µL water droplet was deposited onto the slippery surface with a TA of 20°, which is larger than the SA. Figure 3d shows the water droplet began to slide on the surface after its deposition onto the slippery surface at a 2.0 V applied voltage. After the light was turned on, the water droplet movement was not affected, because the applied voltage does not reach the threshold voltage, corresponding to region I in Figure 3b. When the applied voltage increased to 6.0 V, which belongs to region II in Figure 3b, the water droplet can also slip on the surface after being dropped onto the slippery surface (Figure 3e). After 80 mW cm^−2^ illumination was imposed, the water droplet motion stopped immediately owing to the photoelectric cooperative‐induced enhanced SA. The pinned water droplet instantly resumed sliding on the surface as soon as the light was removed. In addition, patterned writing on slippery surfaces was also achieved utilizing the photoelectric cooperative wetting behavior in region III (8.0–13.0 V) (Figure 3f; Figure S7, Supporting Information). More specifically, when the applied voltage (10.0 V) exceeded the photoelectric‐wetting *V*_ppe_ (*V*_ppe_ is the pinning voltage of ≈8.0 V, at which the water droplets began to be irreversibly pinned on slippery surfaces under 80 mW cm^−2^ illumination) but was lower than the electrowetting *V*_pe_ (*V*_pe_ is the pinning threshold voltage without illumination, ≈13.0 V), under the pull of a “copper pen” with a 10.0 V voltage (copper strip with another end connected to the electrode), the water droplet (2 µL, dyed by methylene blue) can easily slide from the top part of the horizontally placed slippery surface to the bottom sides without any trace (top photographs in Figure 3f). However, when light illumination of 80 mW cm^−2^ was simultaneously applied under an applied voltage (10.0 V), which is larger than *V*_ppe_, photoelectric cooperative infiltration occurred. In this case, the blue water droplet wetted the gap between nanorods and imprinted into the slippery surfaces. Based on this principle, patterned vertical lines (middle photographs in Figure 3f) and letters (bottom photographs in Figure 3f) were successfully written using the “copper pen.” The digital photograph of a vertical line in Figure 3f is indistinct owing to the glaring illumination, and a clear photo without illumination is shown in Figure S7 of the Supporting Information.

To understand the photoelectric cooperative response mechanism, we studied the photoelectric properties of the ZnO‐N3‐FAS film and analyzed the water wettability status and charge distribution on the slippery surfaces. UV–vis diffuse reflectance spectra (**Figure** **4**a) show that the ZnO‐N3 film presented weaker light reflection at 400–800 nm than the ZnO film, showing its strong ability to harvest light in the UV–vis region. A photocurrent action spectrum (Figure 4b) indicated that the ZnO‐N3 film had a photoelectric response in the region of 400–700 nm, indicating that the N3 dye could strongly sensitize the ZnO‐nanorod array film. The Nyquist diagrams of the impedance spectra obtained in the dark and under illumination for the ZnO‐N3 film (Figure 4c) showed that the film had a higher electrical resistance in the dark than under illumination. An energy level diagram and the electron transfer process of the ZnO‐N3 film are presented in Figure 4d. The schematic energy level diagram of the ZnO‐N3 film indicates that the energy bands of N3 and ZnO match.[[qv: 24b,25]] Under illumination, N3 molecules absorb photons from light to generate excitons, which diffuse to the interface and then dissociate into free holes and electrons. Electrons are efficiently transferred from the excited state of N3 to the conduction band of the ZnO layer, ensuring electron transport and resulting in a large increase in the photoconduction of the ZnO‐N3 film. The smart photoelectric response of the ZnO‐N3 film can be attributed to the efficient electron injection from N3 to the vertically aligned ZnO nanorods via highly efficient transport pathways. Consequently, the effective thickness of the dielectric layer decreases,[qv: 25] the threshold voltage drops, and the SA then increases.

**Figure 4:**
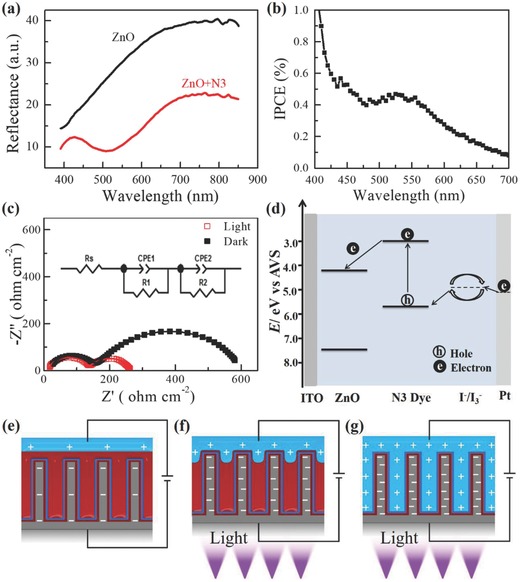
Characterizing properties of ZnO‐N3‐FAS composite films and the photoelectric cooperative response mechanism of a slippery surface: a) UV–vis reflectance spectrum, b) photocurrent action spectrum of the composite film, and c) Nyquist diagrams of the impedance spectra obtained in the dark and under illumination for composite films. d) Schematic energy level diagram of the composite film (the energy level values are referred to the vacuum level). e,f) The water wettability status and charge distribution of the water droplet and the slippery surface at a small applied voltage with and without illumination, and g) photoelectric wetting occurred at high voltage with illumination.

To further confirm the effect of dielectric layer thickness on the threshold voltage, ZnO nanorod arrays with different lengths were prepared for the following experiments (Figures S8 and S9, Supporting Information). Figure S4b,c of the Supporting Information shows that the corresponding water CAs on as‐prepared (uncoated, N3‐coated, N3‐FAS modified) surfaces were 25.3° ± 1.3°,36.2° ± 1.1°, and 136.5° ± 1.9° when the samples were hydrothermally grown for 3.5 h and 20.2° ± 1.4°,30.8° ± 1.5°, and 129.7° ± 1.6° when hydrothermally grown for 12 h, respectively. The modified ZnO‐N3‐FAS surfaces are all suitable (with CAs in region II) for preparing stable photoelectric‐responsive slippery surfaces. The threshold voltage for a ZnO nanorod with a length of 0.83 ± 0.17 µm and a diameter of 62.2 ± 9.2 nm is 2.0 V with light and 4.0 V without light (Figure S10a, Supporting Information), while the threshold voltage for a ZnO nanorod with a length of 2.36 ± 0.15 µm and a diameter of 83.5 ± 8.8 nm is 6.0 V with light and 9.0 V without light (Figure S10b, Supporting Information). The results clearly indicated that as the effective thickness of the dielectric layer decreases, the threshold voltage drops.

To obtain a deep understanding of controllable droplet manipulation on the slippery surface by photoelectric cooperative stimuli, we developed a mechanism using the following aspects: static friction force (*f*), wetting state,[[qv: 3f,9b,26]] and electrostatic interaction. According to the equation of *F*_1_ = *ρgV*sinα and *f* = *μN*, mentioned in our previous work,[[qv: 17b]] a liquid droplet can slide on a surface only when *F*_1_ (parallel component of gravity) ≥*f* (Figure S11, Supporting Information). In our system, *F*_1_ depended completely on α (tilting angle), for *g* (gravitational acceleration) and *V* (volume, 2 µL) were constant, while *f* was closely related to the wetting state (influencing the static friction coefficient, μ),[[qv: 4i,27]] *N* (positive pressure) and electrostatic interactions (generating additional positive pressure) between water and the slippery surface. When an external voltage was applied under dark conditions, a small amount of charge was induced and distributed at the interface of water and the slippery surface, and the water droplet was in a Cassie state (Figure [qv: 4]e) because of the underlying lubrication layer in porous nanogaps.[[qv: 17a,28]] In this case, the weak electrostatic force and the Cassie state implied tiny values of μ and *N*, which produced a corresponding low *f* and SA. When illumination was applied, many electrons and their image charges were generated in the slippery surface and the water droplet, respectively. The increased charge density significantly enhanced the electrostatic force between water and the slippery surface, which allowed water to repel the lubricant (red part) away (Figure [qv: 4]f) and partly intrude into the nanorods space to present a decreased CA (Figure S6a, Supporting Information). The water droplet (blue part) contact state transformed into an unstable Wenzel–Cassie transition state (Figure 4f) with increased substrate coverage, resulting in a large μ and enhanced *f* and SA values.^29^ This labile transition state can evolve into a stable Cassie or Wenzel state when facing an external stimulus. After the illumination was removed, the additional electrostatic attraction between the water droplet and its image charge in the slippery surface disappears instantly. Then, the silicon oil will refill the extruded location from other positions in the slippery surface to induce the contact state to return to the initial Cassie state, endowing droplet motion with a smart switching property. When the voltage was larger than *V*_ppe_ under illumination, photoelectric cooperative wetting occurred, and water fully sink into the space between the nanorods and directly pinned on the ZnO surface (Figure 4g) because the large applied voltage greatly increased the charge density and electrostatic force between water and the slippery surface. In this situation, the water droplet contact state transformed into the pinning Wenzel state, and the SA variations are irreversible.^30^

In conclusion, we successfully obtained a cooperative‐responsive slippery surface based on nanoporous composites. First, the effect of nanoporous composites' wettability on the stability of slippery surfaces was systematically studied. Compared with the results of previously published work on slippery surfaces, we achieved appropriate substrate wetting conditions for preparing a stable slippery surface. Specifically, the water droplets sink into the lubricant layer and contact the substrate with a small CA, making the interface lose its slippery properties; the larger CA of the substrate causes the dewetting of the lubricant fluid on the substrate, which makes it fail to form a slippery surface. Furthermore, controllable droplet motion and patterned writing were realized by photoelectric cooperative stimuli on the slippery surfaces, and the related response mechanism was also deeply studied. This work facilitates an in‐depth understanding of substrate wettability influence on slippery surface stability and their underlying photoelectric cooperative response principles. The work will substantially advance the usage of slippery surfaces in fields such as conductive liquid collection and transfer, microfluidics, microchips, and patterned writing.

## Experimental Section

*Materials and Sample Preparation*: Commercially available zinc acetate (99%, J&K Chemicals, China), 2‐methoxythanol (99%, Sigma‐Aldrich, China), ethanolamine (99%, Sigma‐Aldrich, China), zinc nitrate hexahydrate (99%, J&K Chemicals, China), hexamethylenetetramine (99%, Sigma‐Aldrich, China), *cis*‐bis(4,4′‐dicarboxy‐2,2′‐bipyridine) dithiocyanato ruthenium(II) (N3) (99%, Sigma‐Aldrich, China), 1H,1H,2H,2H‐perfluorodecyltrimethoxysilane (FAS) (99%, J&K Chemicals, China), silicon oil (SD998, Zhongkeshangde, China, viscosity = 40 cSt), carbon black (XFNANO Materials Tech. Co., China), and methylene blue (Sinopharm Chemical Reagent Co., Ltd., China) were all used directly without further treatment. Water was purified using a Milli‐Q purification system (Millipore Corp., Bedford, MA) to give a resistivity of 18 MΩ cm. ITO substrates were sequentially cleaned with detergent, deionized water, ethanol, acetone, and deionized water before being blow‐dried with nitrogen. The aligned ZnO‐nanorod array was prepared using a method similar to a two‐step solution approach reported in the literature.[[qv: 24b]] First, zinc acetate (0.33 g) was added to 2‐methoxythanol (3 g) and stirred at ambient temperature for 15 min. Then, ethanolamine (0.0915) g was added into the abovementioned solution, which was then stirred at room temperature for 2 h to obtain a 0.5 mol L^−1^ crystal seed solution. Next, zinc nitrate hexahydrate (1.19 g) and hexamethylenetetramine (0.56 g) were added to 200 mL deionized water and stirred at room temperature for 2 h, resulting in a 0.02 mol L^−1^ growth solution. Finally, the crystal seed solution was spin‐cast (KW‐4B, China) onto an ITO‐glass substrate two times and annealed at 260 °C to prepare a 100–200 nm thick film. The as‐prepared substrate was suspended in growth solution at 80 °C for different time (3.5, 6, and 12 h) to prepare nanorod samples of different lengths (0.83 ± 0.17, 1.61 ± 0.07, and 2.36 ± 0.15 µm, respectively). Then, they were removed from the solution, rinsed with deionized water and dried at 80 °C for 2 h. N3‐sensitized ZnO‐nanorod array films were prepared by putting the aligned ZnO‐nanorod array films into a 0.05 mmol L^−1^ N3 ethanol solution for 2 h. Then, the films were washed three times using ethanol and subsequently dried at 60 °C for 1 h. Next, the films were treated with different concentrations of FAS ethyl alcohol solution for different time. The water CAs of samples 1–14 and their related modifying conditions (volume ratio of ethyl alcohol to FAS and modifying time) were 84.0° ± 2.2**°** (1:500, 1 h), 94.5° ± 2.4**°** (1:500, 12 h), 105.9° ± 2.2**°** (1:500, 24 h), 115.7° ± 2.0**°** (1:100, 2 h), 122.8° ± 2.1**°** (1:100, 5 h), 123.8° ± 3.7**°** (1:100, 8 h), 125.7° ± 2.3**°** (1:100, 24 h), 131.1° ± 3.1**°** (10:100, 6 h), 137.6° ± 3.2**°** (30:100, 12 h), 140.1° ± 3.2**°** (35:100, 12 h), 144.1° ± 3.3**°** (35:100, 24 h), 148.9° ± 3.9**°** (40:100, 16 h), 150.4° ± 4.9**°** (40:100, 24 h), and 155.0° ± 6.9**°** (50:100, 12 h). Then, the samples were washed several times in ethyl alcohol and subsequently dried at 80 °C for 10 h. All slippery films used in this paper were prepared by immersing the film in silicone oil for 3 min and then holding the film vertically for ≈5 min to remove excess oil.

*Sample Characterization*: SEM images were taken with a JEOL JSM‐7500(Japan), scanning electron microscope with a thin gold coating. XRD patterns were collected by a D/max 2500 X‐ray diffractometer from Rigaku with a Cu Kα radiation. The modification effect of N3 and FAS on ZnO‐nanorod surface were analyzed by SEM‐EDS analysis (JSM‐7500F, JEOL, Japan) at 15 kV. The CAs, SAs, and surface tensions were measured using a Dataphysics OCA20 CA system (Germany) at ambient temperature. A 2 µL water droplet was used in all water‐CA water‐SA measurements. At least five different positions were measured and averaged to obtain a reliable value, using the same sample. Surface tension was calculated through a pendant drop method. A solar simulator (CMH‐250, Aodite Photoelectronic Technology Ltd., Beijing) was used as the light source. The intensity of incident beams was detected via a power and energy meter (Model 1830C, Newport, RI, USA). The applied voltage in the measurement was supplied by a DC power source (HSPY‐120‐02, China) and the corresponding voltages were obtained directly without time steps to achieve more exact data in the experiment. UV–vis reflectance spectra were determined by combining the optic fiber spectrum (NOVA, Idea Optics, Shanghai, China) and the optical microscopy. The incident photon‐to‐current conversion efficiency for solar cells was performed using a commercial setup (PV‐25 DYE, JASCO; a 300 W Xenon lamp was employed as a light source.) The electrochemical impedance measurements were carried out by an electrochemical workstation (CHI660E, China) at room temperature. Silicon oil dyed by carbon black with a concentration of 0.1 g mL^−1^ was dropped onto the composite surface with different surface tension for 30 min and 24 h, and then the related wettability digital photographs were obtained through a Canon EOS 60D camera. Digital photographs of letters written under photoelectric cooperative infiltration using methylene blue aqueous solution (30 mg mL^−1^) as ink were also achieved by the Canon EOS 60D camera.

## Conflict of Interest

The authors declare no conflict of interest.

## Supporting information

Supplementary
