# Supplementary material for: Slippery Surface Based on Photoelectric Responsive Nanoporous Composites with Optimal Wettability Region for Droplets' Multifunctional Manipulation
Source: Adv Sci (Weinh). 2018 Nov 20;6(1):1801231. doi: 10.1002/advs.201801231 (PMC6325596; doi:10.1002/advs.201801231)
Supplement: Supplementary file 1 — Supplementary [file ADVS-6-1801231-s001.pdf]

Copyright WILEY-VCH Verlag GmbH & Co. KGaA, 69469 Weinheim, Germany, 2018.

ADVANCED SCIENCE
Open Access

Supporting Information

for *Adv. Sci.,* DOI: 10.1002/advs.201801231

Slippery Surface Based on Photoelectric Responsive Nanoporous Composites with Optimal Wettability Region for Droplets' Multifunctional Manipulation

Keyu Han, Liping Heng, Yuqi Zhang, Yao Liu, and Lei Jiang*

Copyright WILEY-VCH Verlag GmbH & Co. KGaA, 69469 Weinheim, Germany, 2016.

Supporting Information

Slippery Surface Based on Photoelectric Responsive Nano-Porous Composites with Optimal Wettability Region for Droplets' Multifunctional Manipulation

Keyu Han, Liping Heng*, Yuqi Zhang, Yao Liu and Lei Jiang*

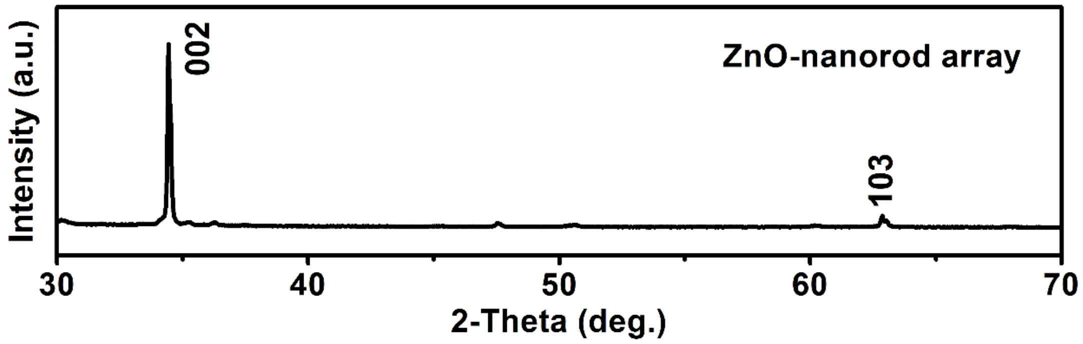

Figure S1. X-ray diffraction (XRD) pattern of ZnO-nanorod arrays grown on indium tin oxide (ITO)-glass substrate with a remarkable (002) peak, which clearly indicates that ZnO (0001) planes are oriented parallel to the basal plane of ITO and that the ZnO nanorods were perpendicularly aligned onto the substrate.

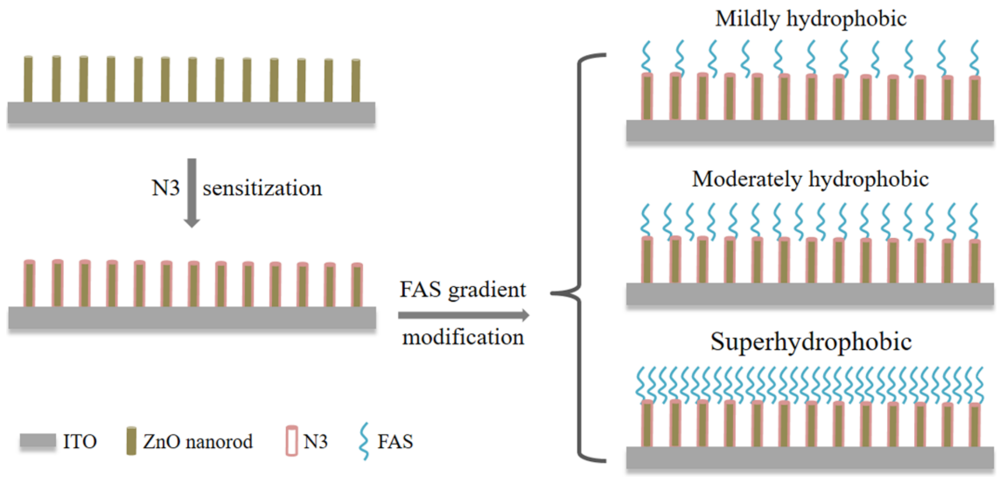

Figure S2. Schematic of ZnO surface sensitization and hydrophobization. The nanorod array surface was first sensitized with the N3 dye and then modified by FAS to achieve different hydrophobic surfaces, such as mildly hydrophobic, moderately hydrophobic and superhydrophobic surfaces. The gradient hydrophobic treatment can be realized by altering

the modifier concentration (FAS ethanol solution) and processing time.

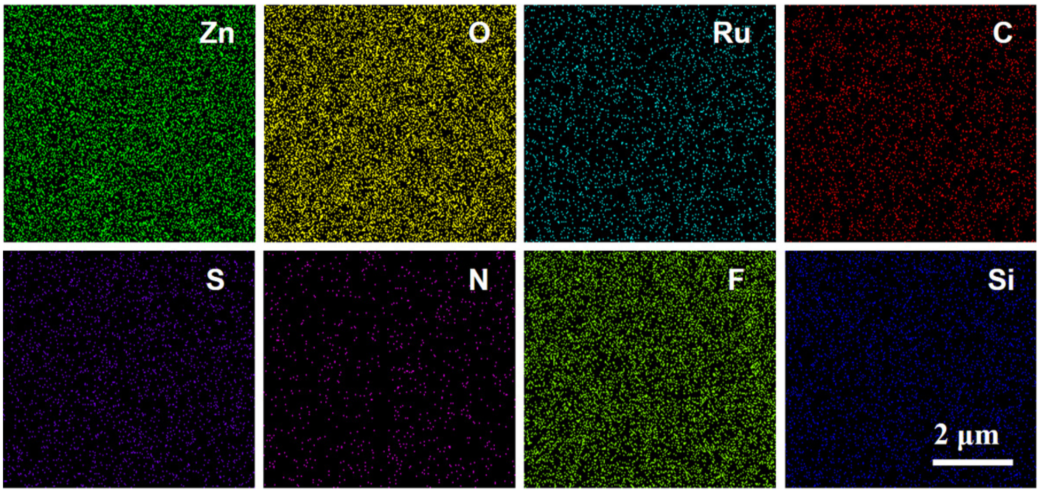

Figure S3. SEM-EDS element analysis of the N3-FAS-modified ZnO nanorod composite surface within the testing area. The results clearly show that the N3 and FAS have been successfully coated onto the underlying ZnO-nanorod surface with uniform distribution.

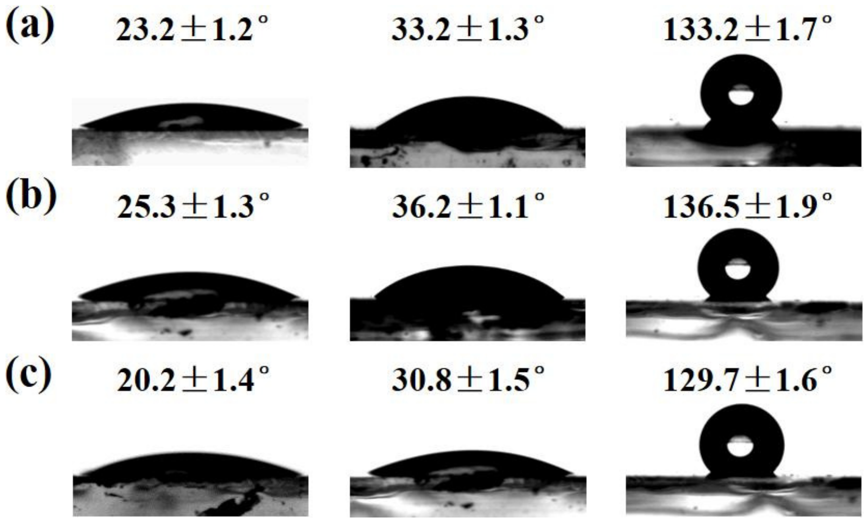

Figure S4. Water CAs on undecorated (left), N3-coated (middle) and N3-FAS-modified (right) ZnO-nanorod surfaces fabricated by hydrothermal growth of different durations. (a) 6 h, with water CAs of 23.2 ± 1.2°, 33.2 ± 1.3° and 133.2 ± 1.7°. (b) 3.5 h, with water CAs of 25.3 ± 1.3°, 36. 2 ± 1.1° and 136.5 ± 1.9°. (c) 12 h, with water CAs of 20.2 ± 1.4°, 30.8 ± 1.5° and 129.7 ± 1.6°. The results show that the N3 sensitization and FAS modification can increase the hydrophobicity and reduce the free energy of the surfaces.

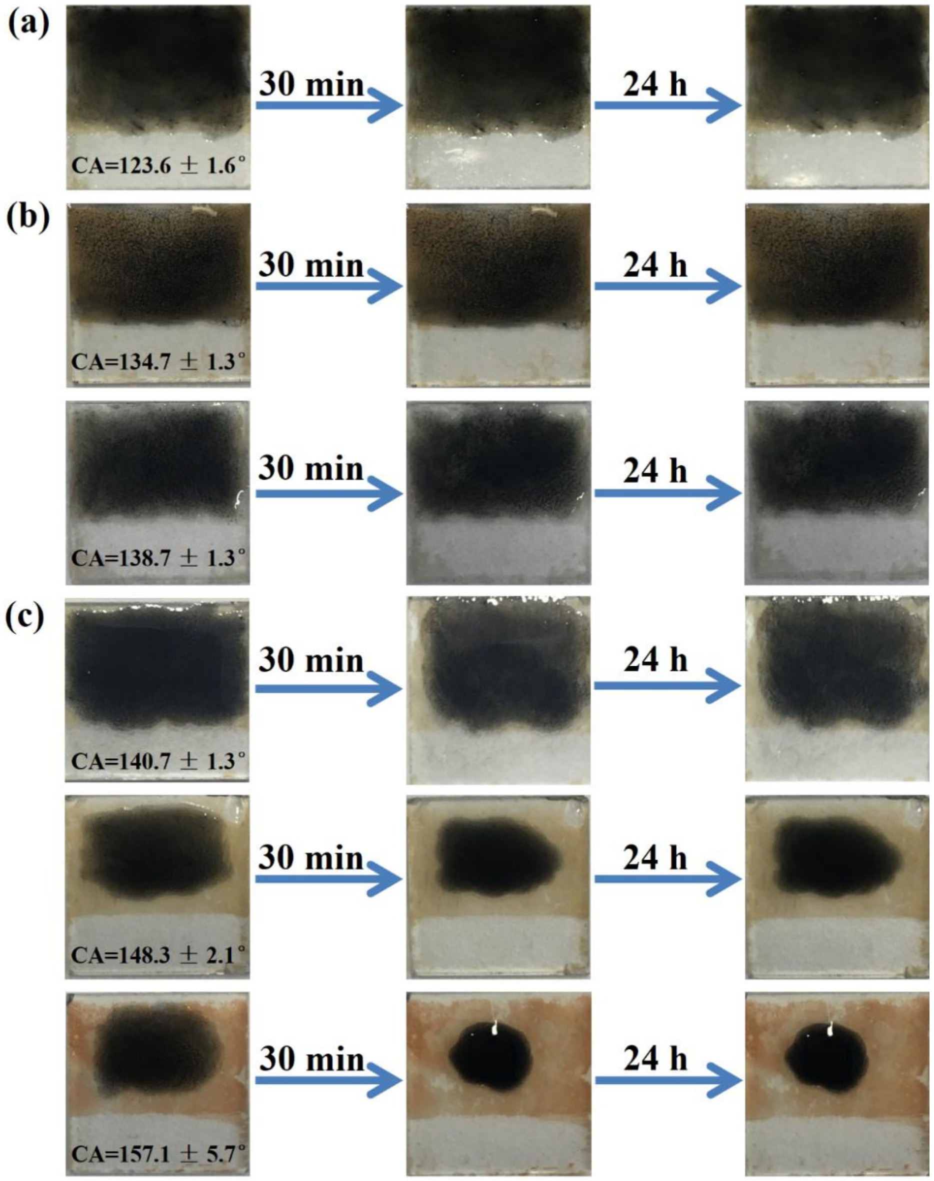

Figure S5. Black-dyed silicon oil forms a stable lubrication layer on the composite surface with a) water CA = 123.6 ± 1.6°, b) water CA = 134.7 ± 1.3° and water CA = 138.7 ± 1.3°. Black-dyed silicon oil cannot form a stable lubrication layer on the composite surface and dewetting occurs with c) water CA = 140.7 ± 1.3°, water CA = 148.3 ± 2.1° and water CA = 157.1 ± 5.7°.

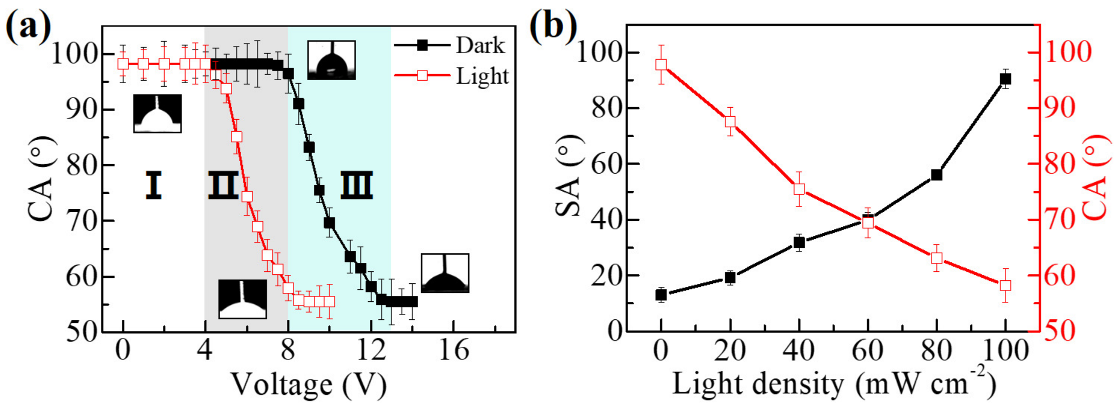

Figure S6. CA and SA responses on the slippery surface with electric fields and photoelectric cooperation stimuli. (a) CA as a function of the applied voltage was measured with (□) and without (■) illumination (the applied light intensity was 80 mW·cm^{-2}). The effects of the applied voltage can be divided into three regions. In region I, the CA does not change with increasing applied voltage, even with illumination; in region II, the CA decreases with increasing applied voltage under light illumination, but it does not change without light illumination; in region III, the CA decreases with increasing applied voltage, even without illumination. (b) CA and SA as a function of the light intensity, measured at an applied voltage of 6.0 V.

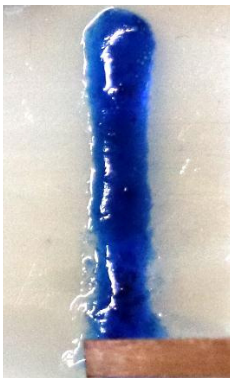

Figure S7. The blue line on the slippery surface written by a "copper pen" through photoelectric cooperative infiltration. The voltage and light intensity used were 10.0 V and 80 mW·cm^{-2}, respectively.

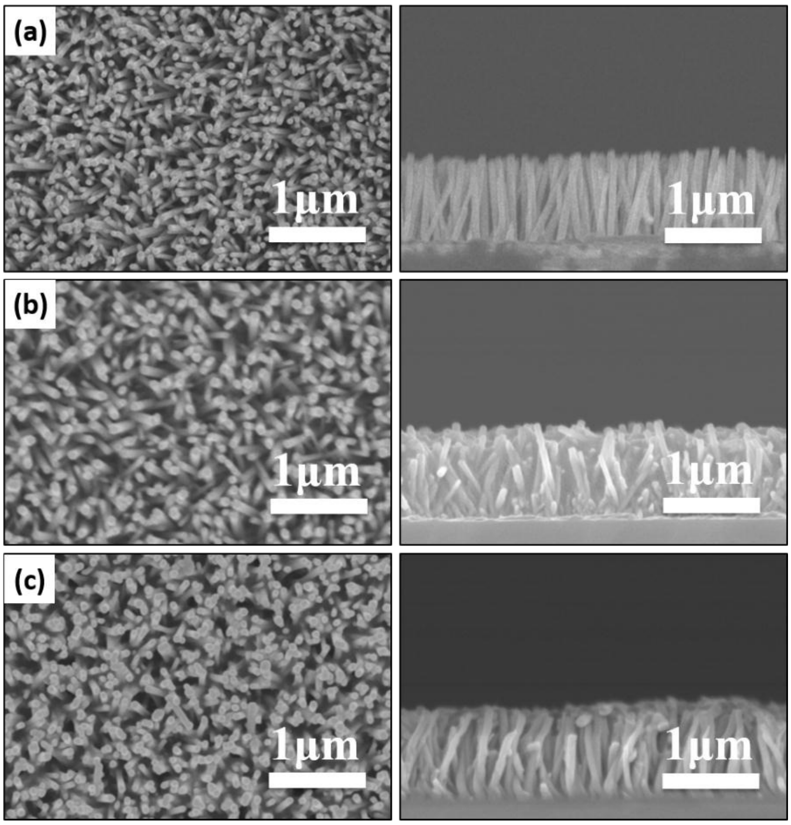

Figure S8. Top-view and side-view SEM images of the as-prepared (a) ZnO nanorod array with diameter of 62.2 ± 9.2 nm, (b) N3-coated ZnO nanorod array with diameter of 64.9 ± 10.9 nm, and (c) N3-coated and FAS-modified ZnO nanorod array with diameter of 72.4 ± 7.6 nm. The nanorod array was prepared by hydrothermally growth for 3.5 h. The side-view SEM images indicate that the nanorods grew almost perpendicularly onto the substrate, with a length of 0.83 ± 0.17 μm. The scale bar is 1 μm.

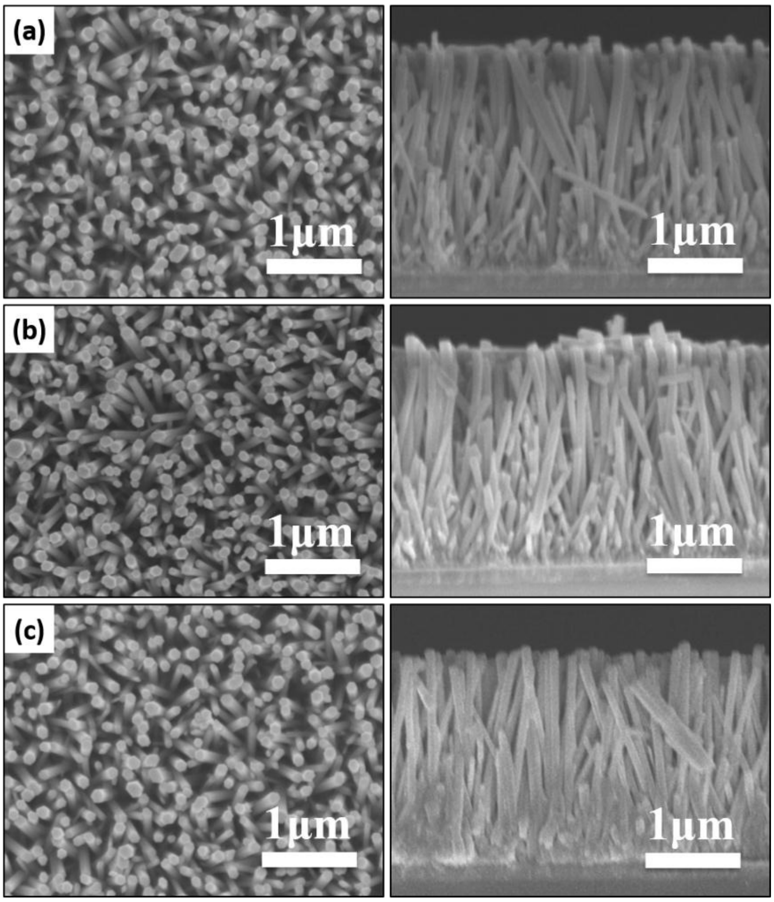

Figure S9. Top-view and side-view SEM images of the as-prepared (a) ZnO nanorod array with diameter of 83.5 ± 8.8 nm, (b) N3-coated ZnO nanorod array with diameter of 84.1 ± 5.9 nm, and (c) N3-coated and FAS-modified ZnO nanorod array with diameter of 89.5 ± 12.2 nm. The nanorod array was prepared by hydrothermally growth for 10 h. The side-view SEM images indicate that the nanorods grew almost perpendicularly onto the substrate with a length of 2.36 ± 0.15 μm. The scale bar is 1 μm.

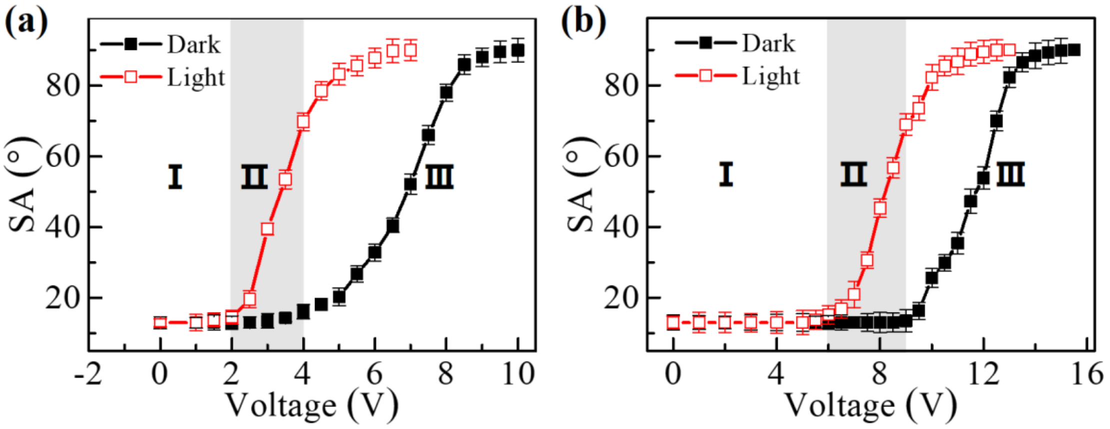

Figure S10. Photoelectric cooperative responsive slippery characteristics of water droplets on the ZnO slippery surfaces with nanorod lengths of (a) 0.83 ± 0.17 μm, (b) 2.36 ± 0.15 μm. SA of the applied voltage was measured with (□) and without (▪) illumination (the applied light intensity was 80 mW·cm^{-2}). The effects of the applied voltage can be divided into three regions. In region I, electrowetting cannot occur, even with illumination; in region II, electrowetting takes place with light illumination; in region III, electrowetting happens, even without illumination.

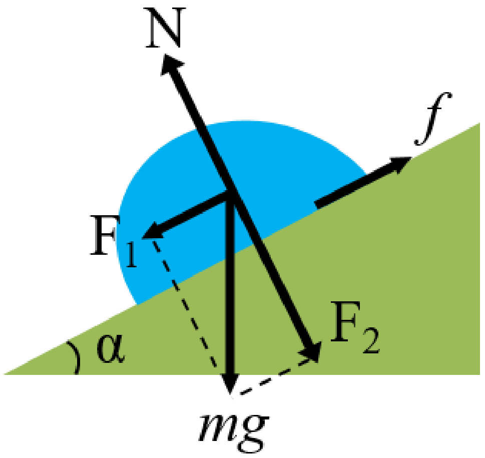

Figure S11. Force analysis of a liquid droplet on a tilted surface.
